# Antennae-abundant expression of candidate cytochrome P450 genes associated with odorant degradation in the asian citrus psyllid, *Diaphorina citri*

**DOI:** 10.3389/fphys.2022.1004192

**Published:** 2022-09-13

**Authors:** Yinhui Kuang, Yu Xiong, Xue Dong Chen, Xiudao Yu

**Affiliations:** ^1^ Ganzhou Key Laboratory of Nanling Insect Biology/Ganzhou Key Laboratory of Greenhouse Vegetables/National Navel Orange Engineering Research Center, College of Life Sciences, Gannan Normal University, Ganzhou, Jiangxi, China; ^2^ Entomology and Nematology Department, University of Florida, Gainesville, FL, United States

**Keywords:** asian citrus psyllid, antennal transcriptome, odorant degrading enzyme, cytochrome P450, gene expression

## Abstract

The Asian citrus psyllid, *Diaphorina citri*, is a notorious pest that is an efficient vector for *Candidatus* Liberibacter asiaticus (*C*Las), the causal agent of citrus huanglongbing (HLB). The olfactory system of insects is crucial for foraging and mating behavior. Antennae-abundant odorant degrading enzymes (ODEs), including cytochrome P450 (CYPs), are important in degrading redundant odorant molecules to recover the insect olfactory. In this study, to isolate the antennal CYP genes of *D. citri*, we generated four transcriptomes from female/male antennae and body through deep sequencing of RNA libraries. Seven *DcCYP* genes preferentially expressed in antennae were first identified by comparing the antennal and body transcriptomes. Phylogenetic analysis grouped four *DcCYPs* (*DcCYP6a13*, *DcCYP6j1*, *DcCYP6k1,* and *DcCYP6a2*) into the CYP3 class, whereas *DcCYP4d2*, *DcCYP4c62,* and *DcCYP4d8* were clustered in the CYP4 clade. qRT-PCR analyses across developmental stages and tissues showed they were antennae-abundant in both genders and constantly expressed from the first instar nymph to the adult. The results presented here highlight the isolation and expression of CYP genes in *D. citri* antennae, providing valuable insights into their putative role in odorant degradation.

## Graphical Abstract

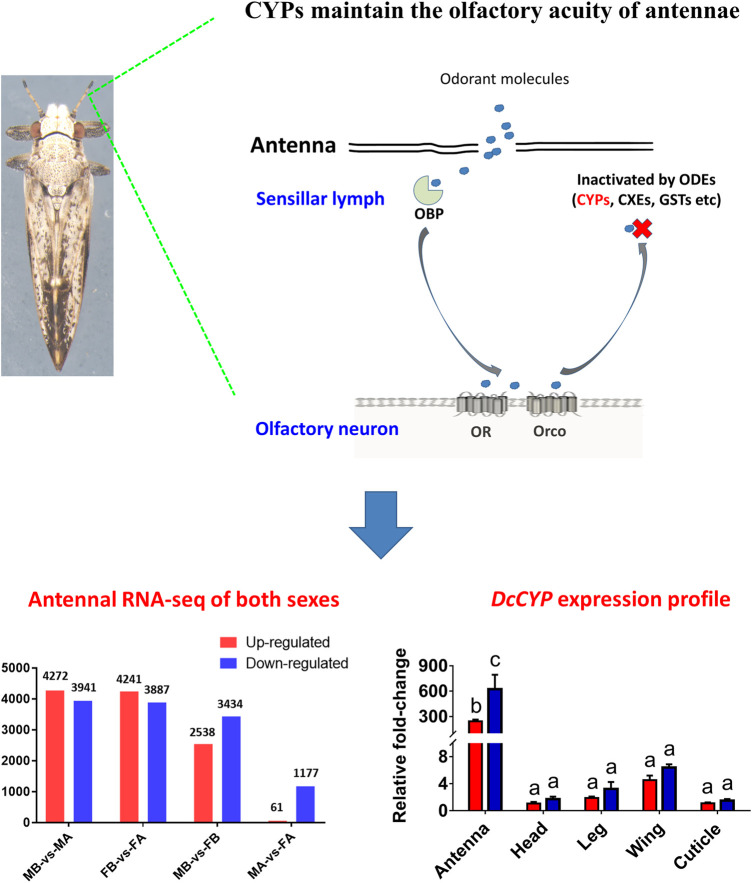

## Introduction

Insect antennae are complex sensory organs that detect various volatile compounds for securing food, mating, and oviposition. The antennal system in insects has been intensively studied using various electrophysiological, molecular, and omics techniques, providing a broad range of biological insights into olfactory perception (Oh et al., 2019; Ahn et al., 2020). The biologic processes of olfactory perception involve interactions between exogenous odor molecules and various families of chemoreception-related proteins, including the odorant-binding proteins (OBPs), chemosensory proteins (CSPs), olfactory receptors (ORs), and odorant-degrading enzymes (ODEs) (Pelosi, 1996; Brito et al., 2016; Fleischer et al., 2018). OBPs and CSPs are small soluble proteins proposed to selectively trap and transport hydrophobic odorant molecules through the sensillum lymph towards the dendrites of olfactory sensory neurons, where ORs are located on the membrane surface (Brito et al., 2016; Cheema et al., 2021; Zhou and Jander, 2022). ORs act as a biotransducer to convert chemical signals into electrophysiological signals. Upon ORs are successfully activated, the odor molecules must be rapidly removed by ODEs, allowing the insect olfactory system to recover and maintain the olfactory acuity (Fleischer et al., 2018; Li F et al., 2018).

ODEs play an integral role in insect chemoreception and they prevent the accumulation of stimulants and subsequent sensory adaptation (Vogt and Riddiford 1981; Younus et al., 2014). Insect ODEs have been mostly known for their involvement in the metabolism of endogenous hormones and steroids, as well as exogenous xenobiotics and allelochemicals (Younus et al., 2014; Blomquist et al., 2021). They include a few antenna-specific or antenna-enriched cytochrome P450 (CYPs), glutathione S-transferases (GSTs), carboxylesterases (CXEs), carboxyl/cholinesterases (CCEs) and UDP-glycosyltransferases (UGTs) (Rogers et al., 1999; Maïbèche-Coisne et al., 2002; Durand et al., 2010a; Durand et al., 2010b; Keeling et al., 2013; Bozzolan et al., 2014; Tan et al., 2014; Younus et al., 2014; He et al., 2015; Li F et al., 2018; Blomquist et al., 2021; Wang et al., 2021; Wei et al., 2021). The olfactory-specific GSTs protect the olfactory system from harmful xenobiotics and inactivate the components of sex pheromone in the *Manduca sexta* and *Bombyx mori* (Rogers et al., 1999; Tan et al., 2014). In the Oriental fruit moth *Grapholita molesta*, four antenna-enriched CXEs are found to modulate foraging and mating behaviors, by hydrolyzing the acetate sex pheromone components (Z/E)-8-dodecenyl and the ester host plant volatiles (Wei et al., 2021).

CYPs represent an important supergene family of detoxification enzymes widely occurring in vertebrates and invertebrates (Nauen et al., 2022). The first antennae-specific CYP, *CYP345E2*, is functionally characterized in the pine beetle *Dendroctonus ponderosae*. This CYP enzyme catalyzes the oxidation of monoterpene pine host volatiles (Keeling et al., 2013). Subsequent studies in *D. ponderosae* document that antennae-abundant *CYP6DE1*, *CYP6DJ1*, *CYP6BW1* and *CYP6BW3*, could oxidize and remove terpenoids from antennae, as well as detoxify host terpenoids to overcome plant defenses, in which some of the terpenoid detoxification products are used as pheromones by both sexes (Chiu et al., 2019a; Chiu et al., 2019b; Chiu et al., 2019c; Blomquist et al., 2021). Examples include antennal *CYP6DE1* catalyzes the conversion of *α*-pinene into trans-verbenol, an aggregation pheromone released by female *D. ponderosae*; *CYP6DJ1* oxidizes terpinolene and limonene to alcohols and an epoxide, *CYP6BW1* and *CYP6BW3* oxidize several diterpene resin acids to cope with host defenses (Chiu et al., 2019a; Chiu et al., 2019b; Chiu et al., 2019c). Recent years, with antennal transcriptome analysis, some antennal CYPs have been identified in *Locusta migratoria* (Wu H et al., 2020) and *Drosophila* (Younus et al., 2014; Baldwin et al., 2021).

The Asian citrus psyllid *Diaphorina citri* Kuwayama serves as an efficient insect vector of huanglongbing (HLB) in citrus production. This disease presents an unprecedented challenge to citrus production by reducing fruit yield and quality (Yu and Killiny, 2020; Zhou, 2020). Foliar application of chemical pesticides is the major strategy for controlling *D. citri*. This is challenged by the fact that resistance development has been reported in many citrus-growing areas (Chen et al., 2018). A potential alternative and effective strategy for insect pest management *via* RNA interfere (RNAi) technique requires the selection of optimal target genes (Yu et al., 2016; Yu and Killiny, 2020). Antennae-abundant ODEs such as CYPs hold a great promise for the potential application of RNAi, in which *DcCYP*-silenced insects are expected to exhibit decreased or disordered mating and foraging behaviors.

The availability of genomic and transcriptomic data facilitated the identification of OBPs and CSPs genes in *D. citri* (Wu et al., 2016; Zhang et al., 2020; Liu et al., 2021). However, to our knowledge, no *DcCYPs* have been reported to contribute to odorant degradation. To identify *DcCYPs* correlated with odorant processing in this study, we performed as follows: 1) a comparative transcriptome analysis of antennae and body from both female and male; 2) isolation and *in silico* analysis of *DcCYPs* abundant in antennae; 3) identification of the expression profile of candidate *DcCYP* genes among tissues and developmental stages.

## Materials and methods

### Insect rearing

A colony of *D. citri* was collected in 2015 using field populations from Nankang District, Jiangxi (Yu et al., 2022). The culture was continuously maintained on *Murraya paniculata* seedlings in insect rearing cages kept at 27–28°C and relative humidity of 60–65% with a 14:10 h light: dark photoperiod.

### Sample preparation and RNA extraction

For antennae collection, adult *D. citri* were collected using an aspirator and anesthetized with CO_2_ for sex separation based on the appearance of their abdomen (Yu and Killiny, 2018). Using fine forceps, a pool of approximately 500 antennae was carefully dissected from males and females, respectively. The whole body of twenty males and females without antennae was prepared as the control. Insect samples were dissected on ice under a stereomicroscope and stored at −80°C until RNA extraction. Total RNAs were isolated from the *D. citri* tissues using Trizol (Sigma, St. Louis, MO, United States), and quantified using a NanoDrop One^C^ spectrophotometer (Thermo Fisher Scientific, MA, United States). The integrity of RNA was assessed on 1% agarose gels.

### cDNA library construction and sequencing

cDNA library construction and Illumina sequencing of *D. citri* samples were performed at Huada Gene Sequencing Center, Wuhan, China. Briefly, Oligo (dT)-attached magnetic beads were used to purify mRNA. Purified mRNA was fragmented into small pieces to synthesize first-strand cDNA using random hexamer-primed reverse transcription, followed by second-strand cDNA synthesis. After end-repair and ligation of adaptors, the products were amplified by PCR. The double-stranded PCR products were heated, denatured and circularized by the splint oligo sequence to create a cDNA library. Pair-end sequencing of the cDNA library was performed on a BGIseq500 platform (BGI-Wuhan, China).

### *De novo* transcriptome data processing

RNA-seq was carried out on pools of antennae and bodies from both sexes with three biological replicates. The sequencing data was filtered to obtain clean reads with SOAPnuke (v1.5.2) by removing low-quality reads (i.e., low-quality base ratio > 20%) and reads with adaptor sequences and/or unknown nucleotides (>5%). Clean reads were aligned to the reference genome [Diaci version 3.0 (Hosmani et al., 2019; Yu et al., 2022)] using HISAT2 (version 2.0.4). Transcriptome *de novo* assembly and functional annotation were conducted in BGI Company as described by Yi et al. (2021). Transcript abundances were calculated by RSEM (version 1.2.12), and differentially expressed genes among samples were determined using DESeq2 (version 1.4.5) with Q value ≤0.05.

### Identification and phylogenetic analysis of antennal *DcCYP* genes

The functional annotation of differentially expressed unigenes or contigs was performed by BLAST algorithm with a cut-off E-value of 10^–5^ in public databases, including NCBI-Nr (http://ftp.ncbi.nlm.nih.gov/blast/db) and Swiss-Prot (http://ftp.ebi.ac.uk/pub/databases/swissprot). The open reading frames (ORFs) of putative antennal *DcCYP* genes were predicted using the ORF finder (http://www.ncbi.nlm.nih.gov/gorf/gorf.html). *DcCYP* sequences were submitted to Pfam database (https://pfam.xfam.org/) and SMART (http://smart.embl.de/) to predict the conserved domain. The number of amino acids, molecular weights (MWs), and theoretical isoelectric points (pIs) of DcCYPs were calculated on ExPASy (http://web.expasy.org/protparam/). The putative N-terminal signal peptides (SPs) of deduced DcCYP proteins were predicted using the SignalP 6.0 server (https://services.healthtech.dtu.dk/service.php?SignalP-6.0). The *DcCYP* genomic sequences were retrieved from citrusgreening.org. The exon-intron structure was determined on the Gene Structure Display Server (GSDS, http://gsds.cbi.pku.edu.cn/) by comparing the full-length ORF sequence with the corresponding genomic DNA sequence. The antennal DcCYPs and CYPs of *Acyrthosiphon pisum* were used for the phylogenetic analysis (Ramsey et al., 2010; Supplementary Table S1). The alignment of CYP protein sequences was performed using CLUSTAL_X (version 1.83). The joint unrooted phylogenetic tree was constructed with MEGA11 using the maximum likelihood method with 1,000 bootstrap replicates (Tamura et al., 2021).

### Quantitative real-time PCR analysis

To examine the expression of *DcCYP* genes, a variety of *D. citri* tissues (antenna, head, leg, wing and cuticle) and samples of *D. citri* at six stages, including the first instar nymph, second instar nymph, third instar nymph, fourth instar nymph, fifth instar nymph, as well as adult male and female were collected separately. First-strand cDNA synthesis was initiated with 500–1,000 ng of purified RNA using the EasyScript^®^ One-Step gDNA Removal and cDNA Synthesis SuperMix (TransGen Biotech, Beijing, China). The synthesized cDNA was stored at −20 C until use.

qRT-PCR reactions were carried out in a 20 μl volume: 4 μl of diluted cDNA, 0.4 μM of each primer and 10 μl of PerfectStart^®^ Green qPCR SuperMix (TransGen Biotech, Beijing, China). All the samples were placed in a Roche LightCycler 96^®^ system (Roche Diagnostics, Mannheim, Germany). Two reference genes, actin (GenBank accession number DQ675553) and GAPDH (GenBank accession number XM_017447140), were used to normalize the amount of cDNA added to the PCR reactions. The relative value of *DcCYP* gene expression was analyzed using the 2^–ΔΔCt^ method (Livak and Schmittgen, 2001). Specific primers for each gene were listed in Supplementary Table S2. Three replicates were performed for each treatment.

### Statistical analysis

To compare the expression level of *DcCYP* gene among different tissues and developmental stages, the ANOVA with Tukey’s multiple comparisons test was performed using GRAPHPAD PRISM version 6.0 (GraphPad Software Inc., La Jolla, CA, United States). A value of *p* < 0.05 was considered statistically significant.

## Results

### Transcriptome sequencing and analysis

**FIGURE 1:**
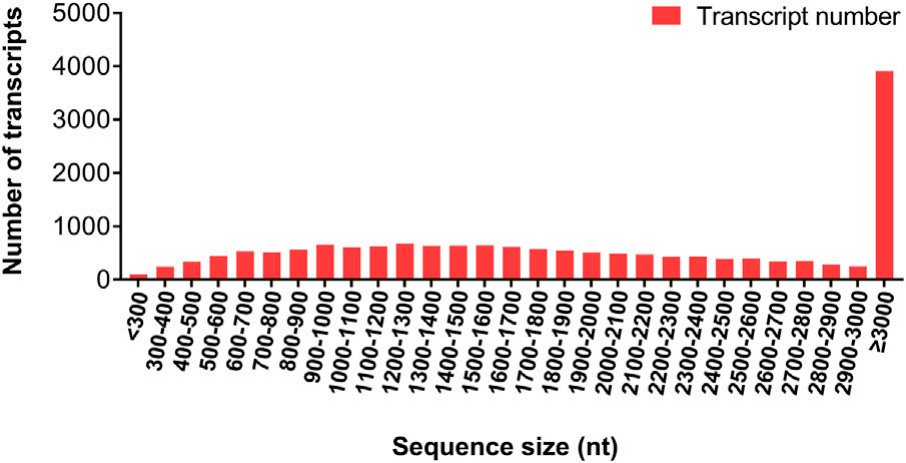
Length distribution of *D. citri* unigenes.

Twelve RNA samples from female antennae (FA), female body (FB), male antennae (MA), and male body (MB) were sequenced using a DNBSEQ platform, with total raw reads of 45.44 M per sample. After data filtering, a total base of 6.46 ± 0.02 Gb per FA, 6.47 ± 0.03 Gb per FB, 6.45 ± 0.02 Gb per MA, and 6.48 ± 0.02 Gb per MB were generated, respectively (Supplementary Table S3). The clean data were subjected to *de novo* transcriptome assembly using Trinity software. A total of 20,520 unigenes were generated, which refer to a uniquely assembled transcript or a cluster of genes that perform a particular function. Among them, 13,827 unigenes (67.38%) were longer than 1,000 bp, and 7,747 (37.75%) were longer than 2000 bp (Figure 1). Additionally, 17,212 (83.88%) unigenes were annotated in at least one public database (e.g. GO, KEGG, KOG, NR, NT, Pfam, and SwissProt databases), while 3,308 (16.12%) unigenes had no matching sequences in any of these databases. All RNA-seq data generated for this study were deposited in the GenBank under Bio-Project accession number PRJNA857870.

### Analysis of differentially expressed genes

**FIGURE 2:**
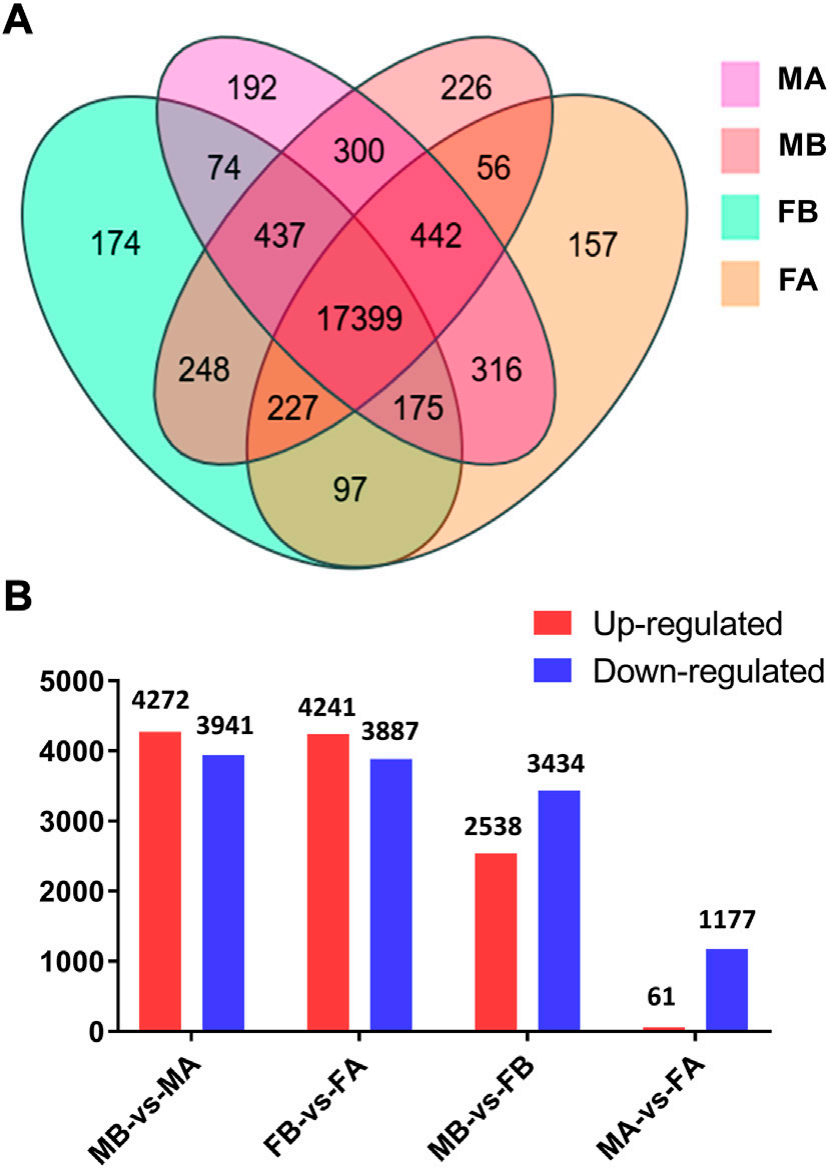
Expression of unigenes in *D. citri* samples. **(A)** Venn diagram of unigenes in the FA, FB, MA and MB of *D. citri*. **(B)** Number of DEGs identified by a pairwise comparison of the four samples. FA, female antennae; FB, female body; MA, male antennae; MB, male body.

Comparative analyses of the antennal and body transcriptomes from both sexes in this study provide useful information to identify the antennae-specific and sex-biased antennal genes. A total of 665 unigenes were detected only in the antenna of females or males; 192 unigenes were unique for MA and 157 were expressed only in FA (Figure 2A). DEGs with a Q value ≤ 0.05 were further identified in antennae, by comparing with the transcriptomes of body tissues. Pairwise comparisons of transcript abundance revealed that 4,241 and 4,272 unigenes were significantly upregulated in FA and MA, respectively (Figure 2B). Twenty DEGs that were most abundant in antennae were listed in Table 1. These included transcripts of odorant binding protein (*DcOBP1*, *DcOBP6*, *DcOBPA10*, and *DcOBP83a*), chemosensory protein (*DcCSP4* and *DcCSP10*), etc. Notably, *DcOBP1*, *DcOBP6*, *DcOBPA10*, *DcOBP83a,* and *DcCSP10* exhibited an antennae-specific expression pattern, in which their antennal FPKM values were >100-fold higher than in the body (Table 1).

**TABLE 1:** Twenty differentially expressed genes (DEGs) that are most abundant in *D. citri* antennae.

Gene ID	Transcriptomic data (mean FPKM)				Gene name
	FA	MA	FB	MB	
Dcitr09g01010.1	156,432 ± 11,677	150,360 ± 8,884	55,317 ± 2,664	53,182 ± 6,217	uncharacterized protein LOC113469368
Dcitr02g18990.1	16,673 ± 1,247	13,986 ± 1,103	5,375 ± 924.1	6,792 ± 891.4	chemosensory protein 4
Dcitr01g13620.1	8,716 ± 2,283	8,272 ± 1,622	13.24 ± 1.546	14.22 ± 0.6486	odorant-binding protein A10
Dcitr01g12900.1	8,008 ± 1,652	7,984 ± 389.5	13.94 ± 0.955	12.67 ± 0.2466	glutathione S-transferase 1
Dcitr01g03170.1	5,667 ± 1,264	5,408 ± 1,185	45.43 ± 4.513	4.223 ± 0.6045	odorant-binding protein 6
Dcitr06g09930.1	5,259 ± 2,255	4,894 ± 1,085	4.897 ± 1.362	6.623 ± 1.925	protein yellow-like
Dcitr01g03210.1	4,862 ± 959.1	4,769 ± 925.6	69.54 ± 9.749	28.81 ± 3.399	odorant binding protein 1
Dcitr13g01140.1	3,803 ± 533.2	3,213 ± 399.1	1,250 ± 57.65	1,289 ± 133.1	no hits
Dcitr04g02650.1	3,262 ± 1755	3,806 ± 1939	291.7 ± 333.5	495.7 ± 768.9	cuticle protein 65
Dcitr12g10130.1	2,911 ± 1,097	2,122 ± 221.7	685.4 ± 80.94	735.7 ± 88.64	titin-like
Dcitr01g15840.1	2,830 ± 306.4	2,251 ± 233.7	1,103 ± 445.5	972.8 ± 117.4	uncharacterized protein LOC103520889
Dcitr08g07580.1	2,546 ± 727.9	2,555 ± 368.3	20.38 ± 3.375	0.2867 ± 0.0472	odorant-binding protein 83a
Dcitr03g06540.1	2,186 ± 674.4	2,751 ± 1,084	654.6 ± 179.3	650 ± 337.7	no hits
Dcitr03g01230.1	1806 ± 986.7	1,537 ± 348.2	0.96 ± 0.5467	0.74 ± 0	no hits
Dcitr08g08410.1	1721 ± 536.5	1,560 ± 63.85	67.66 ± 21.68	73.71 ± 3.957	endocuticle structural glycoprotein SgAbd-2
Dcitr06g07220.1	1,640 ± 92.48	1,637 ± 76.14	427.6 ± 26.3	451.5 ± 16.93	protein takeout-like
Dcitr06g09860.1	1,483 ± 643	1,362 ± 373.4	1.113 ± 0.9026	1.007 ± 0.1747	chemosensory protein 10
Dcitr09g09530.1	1,362 ± 150.7	1,315 ± 250.6	630.8 ± 45.92	832.4 ± 58.85	uncharacterized protein LOC103517165
Dcitr06g09550.1	1,320 ± 9.613	1,209 ± 135.3	673.4 ± 86.43	654.1 ± 11.78	intracellular protein transport protein USO1
Dcitr09g09520.1	1,237 ± 184.2	1,168 ± 32.43	89.18 ± 5.605	86.89 ± 4.627	circadian clock-controlled protein

### Identification of candidate *DcCYP* genes

**FIGURE 3:**
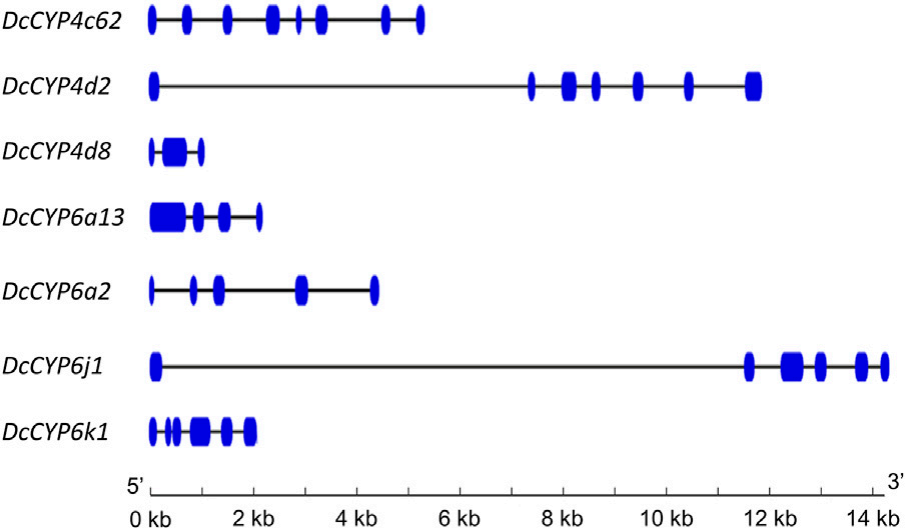
Exon-intron structure of *DcCYP* genes. Exons and introns are indicated by blue boxes and lines, respectively. The length of genes can be inferred by the scale at the bottom.

**FIGURE 4:**
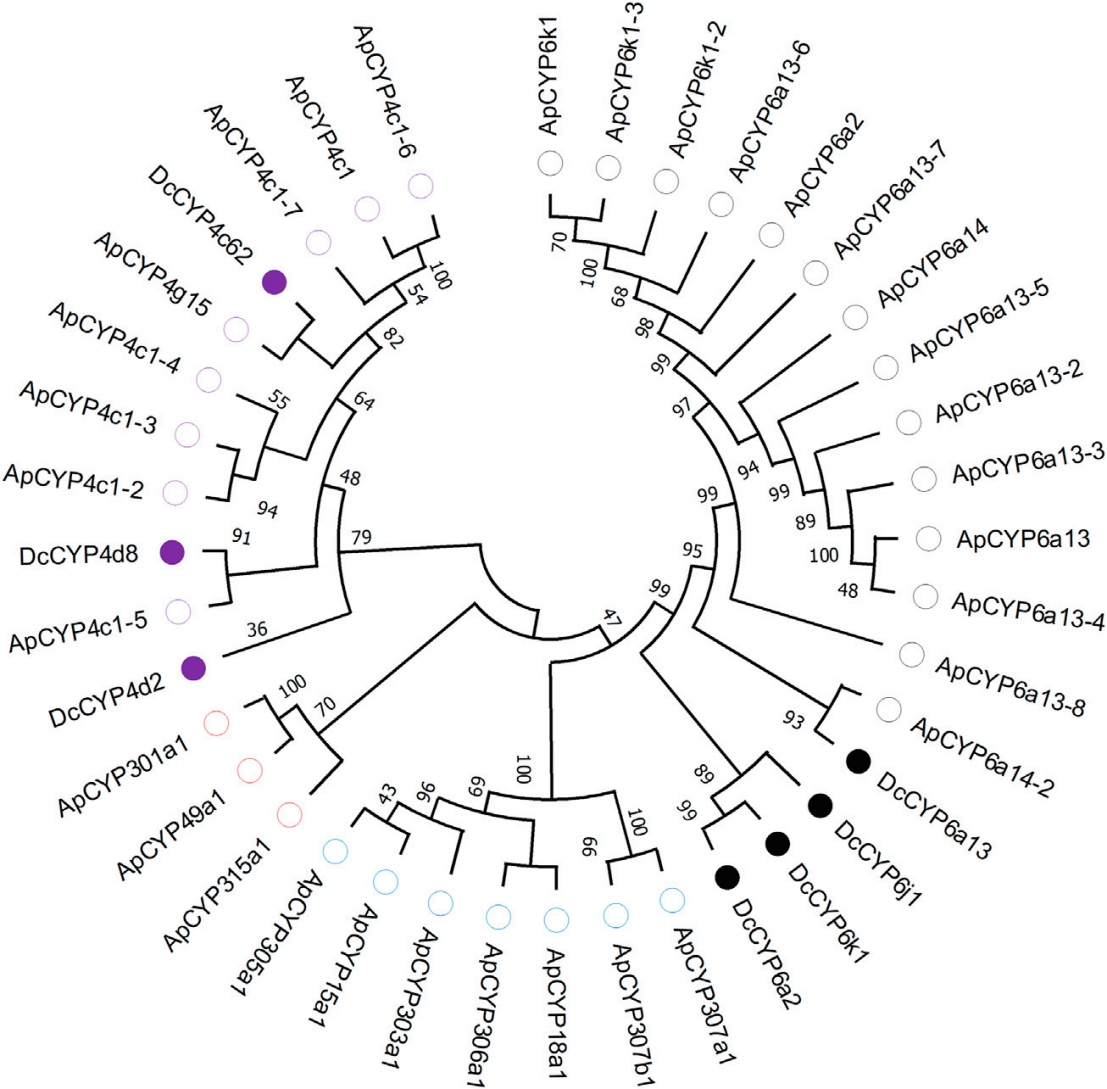
Phylogenetic relationship of 39 CYP proteins from *D. citri* (Dc, 7) and *A. pisum* (Ap, 32). Amino acid sequences were completely aligned using ClustalX 1.83, and a maximum likelihood tree was constructed using MEGA 11. Bootstrap values are shown at the branch points and indicate support as a percentage of 1,000 replicates. Seven *D.* c*itri* CYPs are marked with filled circles. The reference CYPs from *A. pisum* are marked with empty circles. CYPs assigned to CYP2-, CYP3- and CYP4-subclass are labelled by blue, black and purple circles, respectively. Three mitochondrial CYPs are marked by red circles. GenBank accession numbers for the CYP used in phylogenetic analysis are provided in Supplementary Table S1.

Seven DEGs abundant in antennae were identified to be *DcCYPs* by blasting against the Nr database. Their redundancy levels were found to be 15.86–167.70 FPKM in antennae, which were >3-fold higher compared to body group. The predicted Mw was from 28.45 to 60.30 kDa with the predicted pI of 6.14–9.04. All DcCYP proteins harbored a conserved P450 domain (Pfam ID: PF00067), and were predicted to have no signal peptide, indicating their functional location inside the cell (Table 2). Gene structure analysis showed that the length of genomic sequence (1,116–14,366 nt) and the number of introns (2–7) varied dramatically among *DcCYP* genes (Figure 3). A maximum likelihood phylogenetic tree was constructed using the amino-acid sequence of the P450 domain from candidate DcCYPs and *A. pisum* CYPs. Four subclasses (CYP2, CYP3, CYP4, and mitochondrial CYP) were well clustered in relevant phylogenetic branches. *DcCYP6a13*, *DcCYP6j1*, *DcCYP6k1*, and *DcCYP6a2* were categorized in the CYP3 clan; the other three *DcCYP* genes, *DcCYP4d2*, *DcCYP4c62* and *DcCYP4d8* were clustered in the CYP4 class (Figure 4).

**TABLE 2:** Identification of candidate *DcCYP* genes in different expression values (FPKM) in the *D. citri* antennal and body transcriptomes.

Designation	Gene ID	Transcriptomic data (mean FPKM)				ORF (aa)	Mw (kDa)	pI
		FA	MA	FB	MB			
DcCYP4c62	Dcitr05g07670.1	60.71 ± 10.57	56.31 ± 11.59	8.79 ± 1.15	8.62 ± 1.51	509	58.56	6.62
DcCYP4d2	Dcitr01g21810.1	132.80 ± 21.67	167.70 ± 18.98	12.78 ± 0.74	17.07 ± 1.62	513	58.77	8.43
DcCYP4d8	Dcitr13g02280.1	21.53 ± 10.21	15.86 ± 3.69	1.60 ± 0.39	1.90 ± 1.28	249	28.45	6.14
DcCYP6a13	Dcitr02g16350.1	117.60 ± 43.98	103.30 ± 10.93	10.45 ± 0.14	14.39 ± 0.28	436	50.44	8.45
DcCYP6a2	Dcitr11g07150.1	157.70 ± 43.81	140.20 ± 6.02	17.40 ± 2.59	18.53 ± 3.07	298	34.29	8.15
DcCYP6j1	Dcitr06g01770.1	94.21 ± 5.01	94.85 ± 13.64	1.10 ± 0.21	1.38 ± 0.05	514	60.30	8.01
DcCYP6k1	Dcitr03g06130.1	58.92 ± 7.98	49.23 ± 4.27	14.41 ± 2.47	15.61 ± 2.60	449	52.20	9.04

aa, amino acids; Mw, molecular weight; pI, isoelectric points.

### Developmental and tissue expression analysis for *DcCYP* genes

**FIGURE 5:**
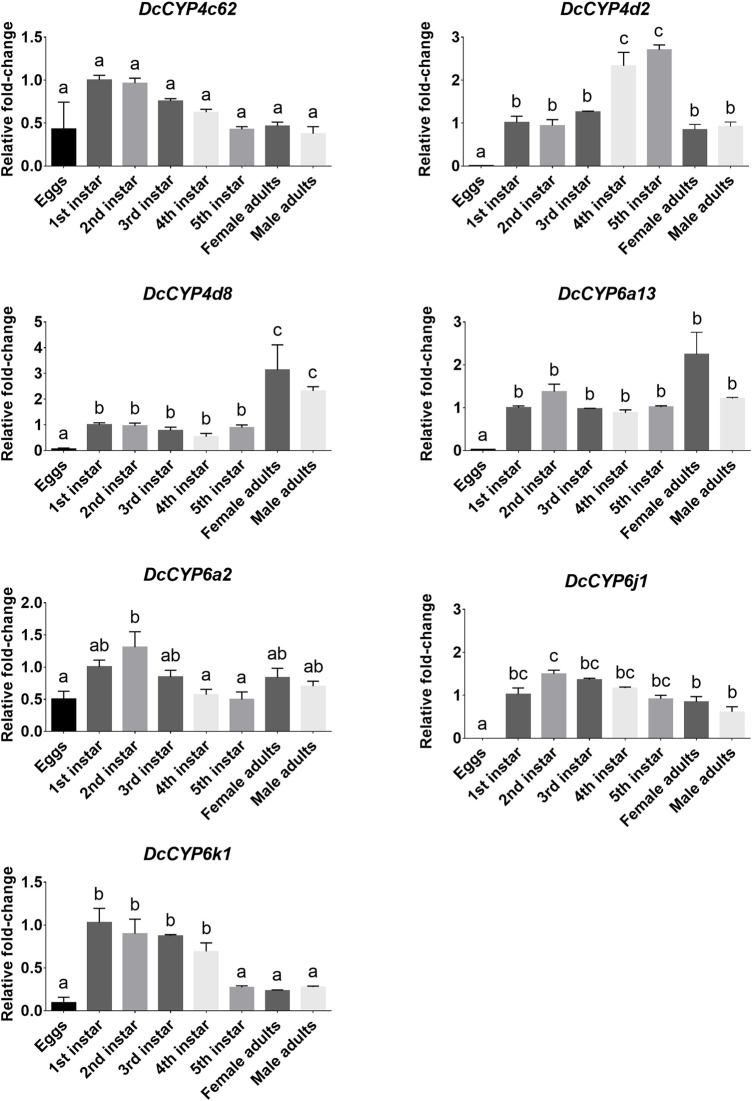
*DcCYP* expression at different life stages of *D. citri*. The expression level of *DcCYP* in first instar nymph was assigned an arbitrary value of 1. The expression levels in other samples are presented relative to the average first instar levels. Error bars represent standard error. Different letters above the error bar indicate significant differences among developmental stages (*p* < 0.05; one-way ANOVA, Tukey’s multiple comparisons test).

**FIGURE 6:**
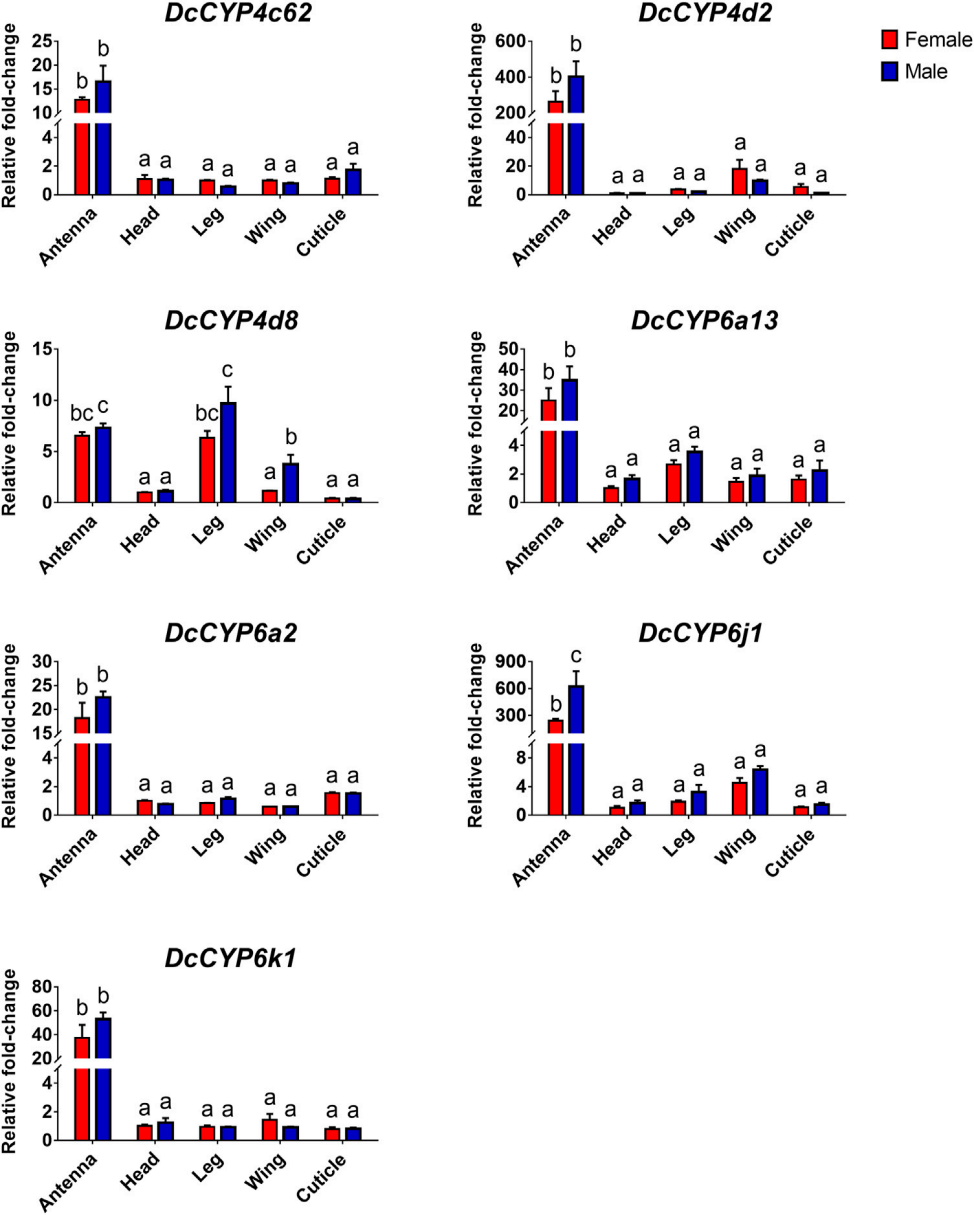
The relative abundance of *DcCYP* transcripts between different sexes and tissues. The expression level of *DcCYP* in female head was assigned an arbitrary value of 1. The expression levels in other samples are presented relative to the average female head levels. Different letters above the error bar indicate significant differences among female and male tissues (*p* < 0.05; two-way ANOVA, Tukey’s multiple comparisons test).

To identify the *DcCYPs* that correlated with odorant degradation in the antennae, *DcCYP* transcripts abundant in the antennae basing on the FKPM methods were selected for developmental and tissue expression analysis. The selected *DcCYP* genes were constantly expressed from the first instar nymph to the adult, along with the low or undetectable levels detected in eggs. However, *DcCYP4d2* transcripts accumulated at a higher level in the last nymphal instar (fourth–fifth), and *DcCYP4k1* exhibited relatively higher expression in the first–fourth nymphal instar (Figure 5). Tissue expression analysis revealed that *DcCYP4c62*, *DcCYP4d2*, *DcCYP6a13*, *DcCYP6a2*, *DcCYP6j1* and *DcCYP6k1* were expressed higher in both male and female antennae than in other non-olfactory tissues, such as heads, legs, wings, and cuticles; *DcCYP4d8* had higher expression levels in the antennae and legs of both sexes (Figure 6). Interestingly, the expression of *DcCYP6j1* calculated by qRT-PCR were a bit higher in the male antennae than in the female antennae, which was inconsistent with the RNA-Seq data (Table 2; Figure 6); this possibly attributed to the difference in sensitivity of two methods.

## Discussion

Chemical control of *D. citri* is threatened by the increased insecticide resistance (Chen et al., 2018). Insect antennae are the main structures responsible for odorant reception *via* sensilla (Ahn et al., 2020; Zhou and Jander, 2022). Identification of antennal ODE such as CYP genes, could provide insights into the odorant recognition mechanism of *D. citri* and further help us to better control this agricultural pest insect. In this study, a total of seven antennae-enriched *DcCYP* genes were first identified by comparing the antennal and body transcriptome data from both sexes. qRT-PCR analyses showed they were antenna-biased and constantly expressed from the first instar nymph to the adult, with *DcCYP6j1* expressed in male antennae at relatively higher levels.

Transcriptome analyses in this study identified several antennae-specific and/or antennae-enriched genes in *D. citri*. As expected, chemosensory gene *OBPs* and *CSPs* were highly expressed in antennae. Among the top twenty DEGs that were abundant in antennae, four transcripts were identified as OBP genes (*DcOBP1*, *DcOBP6*, *DcOBPA10* and *DcOBP83a*), and two DEGs were CSP genes (*DcCSP4* and *DcCSP10*), this was consistent with the putative olfactory role of insect antennae. Antennal OBP and CSP genes have been reported in many other insect species including spotted-wing *drosophila suzukii* (Ahn et al., 2020), ladybird *Aphidius gifuensis* (Kang et al., 2021), as well as hemipteran aphid (Zhou et al., 2010; Wang et al., 2019), hawthorn lace bug *Corythucha ciliata* (Li G. W et al., 2018) and brown plant hopper *Nilaparvata lugens* (Zhou et al., 2014). In green peach aphid *Myzus persicae*, three OBP genes (*MpOBP6/7/10*) were specifically expressed in antennae, and five OBP genes (*MpOBP2/4/5/8/9*) were expressed antennae enriched (Wang et al., 2019).

Insect CYPs perform a variety of important physiological functions, and evidence for their metabolic clearance of plant volatiles or host phytochemical detoxification is accumulating (Blomquist et al., 2021; Vandenhole et al., 2021; Nauen et al., 2022). Insect CYPs could be categorized into CYP2, CYP3, CYP4 and mitochondrial CYP clans (Feyereisen, 2012). Many members of CYP3 and CYP4 clan are currently known or suspected to participate in herbivore adaptation to host plant (Feyereisen, 2012; Blomquist et al., 2021; Vandenhole et al., 2021). For example, the CYP3 P450 genes in *Dendroctonus armandi* and *Oedaleus asiaticus* were shown to be induced by the host terpenoids (pinene and 3-carene) or flavonoid rutin (Dai et al., 2016; Huang et al., 2017); in another study, larval exposure to the plant volatiles induced by *Spodoptera litura* herbivory enhanced transcript levels of CYP3 genes (Sun et al., 2021). From the four clades of P450s commonly found in insects, we found four transcripts (*DcCYP6a13*, *DcCYP6j1*, *DcCYP6k1* and *DcCYP6a2*) were clustered in CYP3 family, and three genes (*DcCYP4d2*, *DcCYP4c62* and *DcCYP4d8*) were grouped in CYP4 clan.

Antennae-abundant CYPs have been functionally demonstrated as ODEs involved in terpenoid detoxification and odorant processing (Keeling et al., 2013; Chiu et al., 2019a; Chiu et al., 2019b; Chiu et al., 2019c; Blomquist et al., 2021). A combined transcriptome and qRT-PCR analysis in this study revealed that seven *DcCYP* genes (*DcCYP4d2*, *DcCYP4c62*, *DcCYP4d8, DcCYP6a13*, *DcCYP6j1*, *DcCYP6k1* and *DcCYP6a2*) were preferentially expressed in antennae. Unexpectedly, six totally different *DcCYP* genes (*CYP3175B1*, *CYP3178A1*, *CYP3640A2*, *CYP4C67*, *CYP380C15* and *CYP3167A7*) were reported to be antennae-abundant in the previous study (Wu Z et al., 2020), by comparing antennal transcriptome with that of gut tissues, reproductive organs, Malpighia tubules, brain, and fat body. However, the selected small tissues and organs are not representatives of non-olfactory tissues, this may contribute to the conﬂicting results of previous studies. In addition, the expression of *DcCYP6j1* was observed to be a bit higher in male antennae. Our results, together with previous findings that male-biased CYPs were usually implicated in the recognition processes of sex pheromone (Maïbèche-Coisne et al., 2004; Feng et al., 2017), suggesting a putative role of these *DcCYPs* in inactivation of plant volatile semiochemicals and/or sex pheromone. Future research elucidating their physiological role will help understand the olfactory mechanism of hemipteran insect, and offer new targets for developing behavioral interference control strategy against *D. citri*.

## Data Availability

The datasets presented in this study can be found in online repositories. The names of the repository/repositories and accession number(s) can be found below: https://www.ncbi.nlm.nih.gov/, PRJNA857870.
