# Supplementary material for: Antennae-abundant expression of candidate cytochrome P450 genes associated with odorant degradation in the asian citrus psyllid, Diaphorina citri
Source: Front Physiol. 2022 Sep 13;13:1004192. doi: 10.3389/fphys.2022.1004192 (PMC9513247; doi:10.3389/fphys.2022.1004192)
Supplement: Supplementary file 1 [file Table1.DOCX]

Supplementary Material

**Supplementary Table 1.** Proteins used for phylogenetic analysis

| CYP Protein | Species | GenBank No. |
| --- | --- | --- |
| DcCYP6a13 | *Diaphorina citri* | XP_026681205 |
| DcCYP6j1 | *Diaphorina citri* | XP_008477728 |
| DcCYP4d2 | *Diaphorina citri* | QBQ34514 |
| DcCYP6k1 | *Diaphorina citri* | XP_008479288 |
| DcCYP4c62 | *Diaphorina citri* | QBQ34519 |
| DcCYP4d8 | *Diaphorina citri* | XP_008477747 |
| DcCYP6a2 | *Diaphorina citri* | XP_026681602 |
| ApCYP306a1 | *Acyrthosiphon pisum* | XP_001947874 |
| ApCYP15a1 | *Acyrthosiphon pisum* | XP_001952620 |
| ApCYP305a1 | *Acyrthosiphon pisum* | XP_001950295 |
| ApCYP307a1 | *Acyrthosiphon pisum* | XP_001945761 |
| ApCYP18a1 | *Acyrthosiphon pisum* | XP_001947923 |
| ApCYP303a1 | *Acyrthosiphon pisum* | XP_001951093 |
| ApCYP307b1 | *Acyrthosiphon pisum* | XP_001948715 |
| ApCYP6k1 | *Acyrthosiphon pisum* | XP_001943150 |
| ApCYP6a13 | *Acyrthosiphon pisum* | XP_001948443 |
| ApCYP6a13-2 | *Acyrthosiphon pisum* | XP_001946384 |
| ApCYP6k1-2 | *Acyrthosiphon pisum* | XP_001945833 |
| ApCYP6a13-3 | *Acyrthosiphon pisum* | XP_001943570 |
| ApCYP6a13-4 | *Acyrthosiphon pisum* | XP_001948581 |
| ApCYP6a13-5 | *Acyrthosiphon pisum* | XP_001951466 |
| ApCYP6a14 | *Acyrthosiphon pisum* | XP_001945100 |
| ApCYP6a13-6 | *Acyrthosiphon pisum* | XP_001943981 |
| ApCYP6a2 | *Acyrthosiphon pisum* | XP_001947920 |
| ApCYP6k1-3 | *Acyrthosiphon pisum* | XP_001948421 |
| ApCYP6a13-7 | *Acyrthosiphon pisum* | XP_001952450 |
| ApCYP6a13-8 | *Acyrthosiphon pisum* | XP_001946428 |
| ApCYP6a14-2 | *Acyrthosiphon pisum* | XP_001944599 |
| ApCYP4c1 | *Acyrthosiphon pisum* | XP_001943923 |
| ApCYP4c1-2 | *Acyrthosiphon pisum* | XP_001951034 |
| ApCYP4c1-3 | *Acyrthosiphon pisum* | XP_001952439 |
| ApCYP4g15 | *Acyrthosiphon pisum* | XP_001944205 |
| ApCYP4c1-4 | *Acyrthosiphon pisum* | XP_001944051 |
| ApCYP4c1-5 | *Acyrthosiphon pisum* | XP_001948141 |
| ApCYP4c1-6 | *Acyrthosiphon pisum* | XP_001944092 |
| ApCYP4c1-7 | *Acyrthosiphon pisum* | XP_001952110 |
| ApCYP315a1 | *Acyrthosiphon pisum* | XP_001944183 |
| ApCYP49a1 | *Acyrthosiphon pisum* | XP_001946744 |
| ApCYP301a1 | *Acyrthosiphon pisum* | XP_001948959 |

**Supplementary Table 2.** Oligonucleotide primer pairs used in this study

| Gene | Primer name | Sequences of primers (5′→3′) | Application |
| --- | --- | --- | --- |
| *DcCYP6a13* | DcCYP6a13 F | CAAGATGAGCTACCTGGATAAGG | qRT-PCR |
|  | DcCYP6a13 R | GTGTTGGGCAGCTTGTATTTC |  |
| *DcCYP6j1* | DcCYP6j1 F | GCACTCACTACCACGTTCTT | qRT-PCR |
|  | DcCYP6j1 R | CGCTCCATCCTCCTTAGATTTC |  |
| *DcCYP4d2* | DcCYP4d2 F | GCCGGACAAGATACCACTAAA | qRT-PCR |
|  | DcCYP4d2 R | GCACACGGTCAATCTCTTCT |  |
| *DcCYP6k1* | DcCYP6k1 F | GCACTTATCCAGCTCTCCTAATC | qRT-PCR |
|  | DcCYP6k1 R | TCACTGTCAGACACATGGAAC |  |
| *DcCYP4c62* | DcCYP4c62 F | CACAGATGTGCAGACCAGTATC | qRT-PCR |
|  | DcCYP4c62 R | GCACTGAACGGTATGTAGGAATAG |  |
| *DcCYP4d8* | DcCYP4d8 F | GACAGTTGTATCTGCGGGTTA | qRT-PCR |
|  | DcCYP4d8 R | CGATCTCATCGTAGACCTCTTG |  |
| *DcCYP6a2* | DcCYP6a2 F | GCCGTACACTTCACTCTCTTC | qRT-PCR |
|  | DcCYP6a2 R | GCCTTCTCTTGTTTCCTCATCT |  |
| *DcActin* | DcActin F | AGAAAGTACTCCGTGTGGATTG | qRT-PCR |
|  | DcActin R | CGGACTCGTCGTATTCTTGTT |  |
| *DcGAPDH* | DcGAPDH F | TGAGATCAAGGCCAAGGTAAAG | qRT-PCR |
|  | DcGAPDH R | GTCAAAGATGGAGGAGTGAGTG |  |

**Supplementary Table 3.** Summary of the transcriptome data of antenna and body between the sexes of *D. citri*

| Samples | Total Raw Reads (M) | Total Clean Reads (M) | Total Clean Bases (Gb) | Clean Reads Q20 (%) | Clean Reads Q30 (%) | Clean Reads Ratio (%) |
| --- | --- | --- | --- | --- | --- | --- |
| FA1 | 45.44 | 43.22 | 6.48 | 96.71 | 90.7 | 95.13 |
| FA2 | 45.44 | 43.12 | 6.47 | 96.7 | 90.69 | 94.89 |
| FA3 | 45.44 | 42.93 | 6.44 | 96.66 | 90.57 | 94.47 |
| FB1 | 45.44 | 42.92 | 6.44 | 96.55 | 90.27 | 94.45 |
| FB2 | 45.44 | 43.20 | 6.48 | 96.41 | 89.89 | 95.07 |
| FB3 | 45.44 | 43.30 | 6.50 | 96.46 | 90.00 | 95.30 |
| MA1 | 45.44 | 43.03 | 6.45 | 96.69 | 90.61 | 94.70 |
| MA2 | 45.44 | 42.89 | 6.43 | 96.47 | 90.02 | 94.39 |
| MA3 | 45.44 | 43.15 | 6.47 | 96.69 | 90.66 | 94.97 |
| MB1 | 45.44 | 43.33 | 6.50 | 96.38 | 89.81 | 95.35 |
| MB2 | 45.44 | 43.03 | 6.46 | 96.4 | 89.88 | 94.71 |
| MB3 | 45.44 | 43.17 | 6.48 | 96.42 | 89.94 | 95.01 |
